# Impact of literature reports on drug safety signals

**DOI:** 10.1007/s00508-020-01677-y

**Published:** 2020-05-26

**Authors:** Bartlomiej Ochyra, Maciej Szewczyk, Adam Przybylkowski

**Affiliations:** 1 Department of Gastroenterology and Internal Diseases, Medical University of Warsaw, Banacha 1a, 02-097 Warsaw, Poland; 2 MS Pharm s.r.o., Praga, Czech Republic

**Keywords:** Drug safety evaluation, Literature search, Risk-benefit analysis, Bibliographic databases, Signal management

## Abstract

**Background:**

Signal management is considered an important activity in pharmacovigilance and should be performed using any available source of data, including scientific literature. The main aim of this study was to assess the role of scientific literature in both indexed and unindexed journals and compare the relevance of both in the signal management process.

**Methods:**

The study was a retrospective analysis of safety data. For the purposes of the study, drugs for which safety signals were evaluated by European Medicine Agency (EMA) were chosen. A match analysis of data collected in the EudraVigilance (EV) database with data from bibliographic databases such as MEDLINE, Embase or EBSCO (International Pharmaceutical Abstracts, IPA and the Allied and the Complementary Medicine Database, AMED) was performed.

**Results:**

A total of 73 drug event associations (DEA) and 4160 individual case safety reports (ICSRs) were analyzed. About 33% of ICSRs were created based on scientific literature. A total of 1196 ICSRs were submitted to the EV database based on journals indexed in global bibliographic databases Embase (86.00%) or MEDLINE (81.96%) or EBSCO (IPA or AMED, 0.66%).

**Conclusion:**

This study underlines the importance of scientific literature for the signal management process in addition to other data sources. Most literature ICSRs from this analysis were created based on scientific journals indexed in bibliographic databases; therefore, it can be concluded that a systematic review of bibliographic databases, such as Embase or MEDLINE is highly relevant for the signal management process.

## Introduction

Signal management is considered as an important activity in pharmacovigilance [1, 2]. Referring to Regulation (EU) No 520/2012 of 19 June 2012 the signal management process shall include the following activities: signal detection, signal validation, signal confirmation, signal analysis and prioritization, signal assessment, and recommendations for action [3].

Drug safety is assessed during both premarketing and postmarketing phases. Signal detection is a key step of the process, and a fundamental part of the postmarketing drug surveillance system [4]. The primary goal of this system is the timely detection of new and/or changed risks associated with an active substance or a medicinal product, for example a new adverse drug reaction (ADR) [5, 6]. A new aspect of a known risk (including known ADR) may change in severity, frequency, ADR outcome, duration, distribution (e.g. gender, age and country) and clinical nature [1].

Signals can arise from a wide variety of data sources. This includes all scientific information concerning the use of medicinal products and the outcome of the use, i.e. quality, non-clinical and clinical data (including pharmacovigilance and pharmacoepidemiological data). Signal detection should be performed using any available source of data, including data from the scientific literature [1].

The relevance of literature review in signal detection has been confirmed in many studies. For example, Klose et al. presented a relationship between floppy iris syndrome and tamsulin. The authors informed that during the same time period 13 cases were published in the literature, but none were spontaneously reported [5]. Such differences can be related to difficulties in identification and correct classification of some ADRs by consumers/patients. Based on the above example, it can be concluded that some safety concerns have little chance of detection based on spontaneous reports; however, it seems that healthcare professionals are more interested in publishing such reactions than reporting them spontaneously to the health authorities (HA) or marketing authorization holder (MAH) [7].

The literature is the source of individual case safety reports (ICSRs) and articles containing relevant safety information for inclusion in periodic safety update reports (PSUR) and/or emerging safety issue processes [8, 9]. Many journals are not indexed in the most popular bibliographic databases. Information published in these journals can be a source of significant information relevant for the assessment of the risk-benefit balance. Because of that according to the guideline on good pharmacovigilance practices (GVP): Module VI, the MAH is expected to perform a systematic review of widely used reference databases and monitor scientific publications in local journals in countries where the medicinal products have marketing authorization [8].

Based on best knowledge, there has been no previous research comparing the relevance of the systematic review of journals indexed in bibliographic databases and unindexed journals for the signal management process. The main aim of this study was to assess the role of scientific literature in both indexed and unindexed journals and compare the relevance of both in the signal management process.

## Method

The study was a retrospective analysis of publicly available data. For the purposes of the study, drugs for which safety signals were evaluated by the European Medicine Agency (EMA) between January 2016 and September 2018 were chosen. Only signals that led to product information (summary of product characteristics, SmPC and/or patient information lLeaflet, PIL) updates were analyzed. The combination of adverse event and drug was classified as a drug event association (DEA) and used for further analysis.

The relevant Pharmacovigilance Risk Assessment Committee (PRAC) recommendations on safety signals published on the EMA website (www.ema.europa.eu) were used to obtain a list of relevant drug international nonproprietary names (INNs) and the Medical Dictionary for Regulatory Activities (MedDRA) terms for each DEA. Preferred terms (PTs) for all signals were matched based on MedDRA version 21.1 for the purpose of line listing reports downloading process (line listing report provides listing of ICSRs for a specific substance and specific MedDRA terms).

As the next step, line listings were downloaded from the EudraVigilance (EV) database via the www.adrreports.eu website. The line listings were the source of data used for the assessment of the literature ICSR created based on both indexed and unindexed journals. The reference period for line listing (start date and end date of the period for reports taken into consideration during the analysis) was defined based on the EV gateway receipt date. The start date was specified as the date of first report for DEA identified in EV. The end date of the reference period was specified as the last day of the relevant PRAC meeting. A duplicate check process was performed to exclude possible duplicates.

A total of four bibliographic databases were selected for verification of literature ICRS: Embase, MEDLINE, EBSCO. The EBSCO covers a wide variety of resources, nevertheless only International Pharmaceutical Abstracts (IPA) and the Allied and the Complementary Medicine Database (AMED) were analyzed to imitate the process of medical literature monitoring performed by the EMA.

For each literature ICRS obtained from a line listing, these databases were analyzed to assess whether the journal which published a specific literature ICRS is indexed in the relevant database. During the next step, the collected data were analyzed using descriptive analysis.

## Results

### Basic characteristics of analyzed DEAs

In the period studied 82 DEAs were identified. For nine DEAs, there was no possibility to download relevant data from EV due to lack of data or problems with matching the proper PTs and/or INNs relevant for download of line listing. Therefore 73 DEAs were analyzed. The analyzed DEAs belonged to 12 different groups according to the anatomical therapeutic chemical (ATC) classification system.

### Impact of data from scientific literature on signal management

For the 73 analyzed DEAs, there were 4160 ICSRs retrieved from the EV. About 33% of ICSRs originated from scientific literature (Table 1). For 13 DEAs there was no literature ICSRs. For 2 DEAs literature ICSRs constituted 100% of ICSRs submitted to the EV database during the analyzed period of time.

**Table 1:** Source of individual case safety reports

Data source	Number of ICSRs
Scientific literature (excluding scientific meetings)	1206
Scientific meetings	157
Other spontaneous (excluding scientific literature and scientific meetings)	2796

*ICSR* individual case safety report

The highest number of literature ICSRs (499; 62.45%) were reported for anti-infective drugs, while the lowest number of literature ICSRs (1; 20%) was noted for musculoskeletal system drugs.

The longest period between EV gateway receipt date (date of case receipt in EV) of first literature ICSR describing an analyzed adverse event and date of the first discussion at a PRAC meeting was 13 years. The shortest period between EV gateway receipt date of first literature ICSR describing an analyzed adverse event and signal discussion was 53 days.

**Fig. 1:**
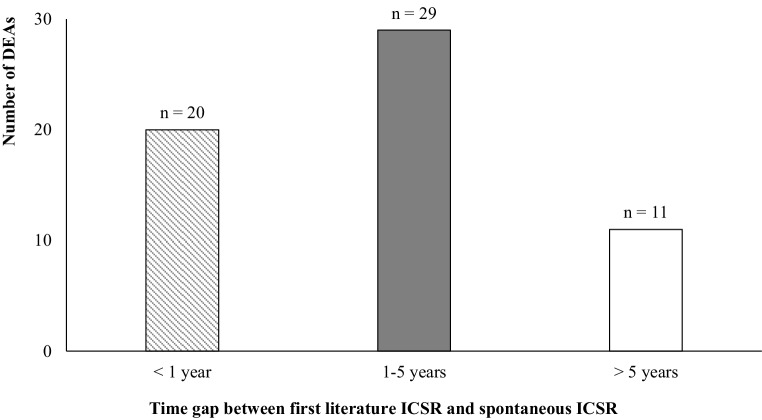
Time gap between first literature individual case safety report (ICSR) and spontaneous ICSR report. *DEA* drug event association

For 12 DEAs the first ICSR was a literature ICSR. For 11 DEAs the first literature ICSR was indexed in Embase and MEDLINE, for 1 DEA the first literature ICSR was indexed only in Embase. Only for 1 DEA was the first literature ICSR not indexed in the analyzed bibliographic databases. The average delay between first ICSR from a source other than the literature and first literature ICSR was 38 months (Fig. 1). For 61 DEAs the first ICSR was a spontaneous report (other than the literature).

### Impact of the analyzed bibliographic database on signal management

**Fig. 2:**
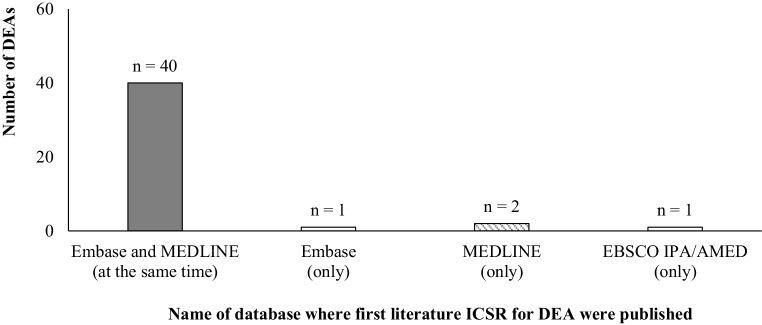
Database of first literature individual case safety report (ICSRs) for drug event association (DEA). *IPA* international pharmaceutical abstracts, *AMED* the allied and the complementary medicine database

Of all literature ICSRs 1206 (88.42%) were created based on scientific journals indexed in Embase or MEDLINE or EBSCO (IPA/AMED). The highest number of ICSRs were created based on scientific journals indexed in Embase. The lowest number of ICSRs were created based on scientific journals indexed in EBSCO (IPA/AMED) (Table 2). First literature ICSR for DEA was mainly published in Embase and MEDLINE (at the same time). Only for 1 DEA was the first ICSR submitted to EV created based on literature not indexed in any database (Fig. 2).

**Table 2:** Indexation databases of literature individual case safety reports (ICSRs)

Database	Number of ICSRs
Embase	1173
MEDLINE	1118
EBSCO (IPA and AMED databases)	9
Only in Embase	79
Only in MEDLINE	24
Only in EBSCO (IPA and AMED databases)	9

*DEA* drug event association, *IPA* international pharmaceutical abstracts, *AMED* the allied and the complementary medicine database

### Primary source country of unindexed literature ICSRs

**Fig. 3:**
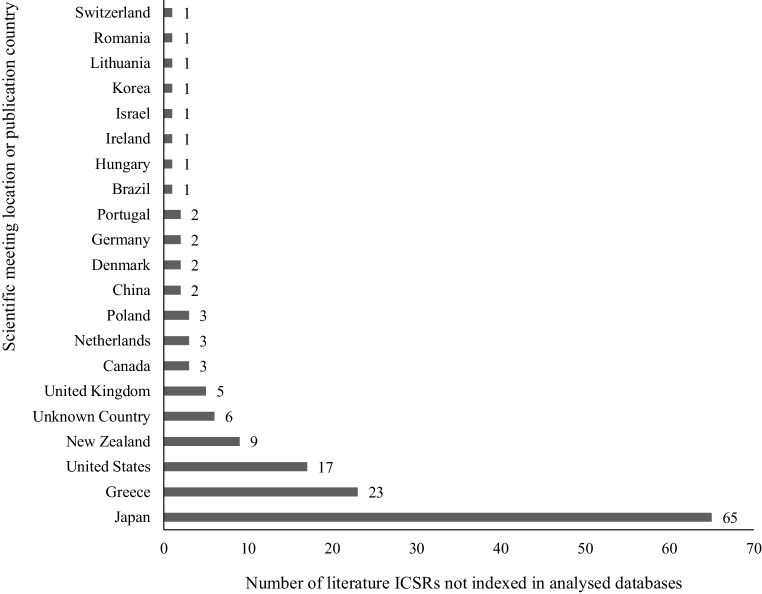
Scientific meeting location or publication country of literature individual case safety report (ICSRs) not indexed in Embase, MEDLINE, EBSCO (IPA/AMED)

The highest amount of unindexed literature ICSRs originated from Japan (65 ICSRs; 43.33%), Greece (23 ICSRs; 15.33%) and the USA (17 ICSRs; 11.33%) (Fig. 3).

## Discussion

About 33% of ICSRs from this analysis originated from scientific literature. For 3 DEAs in this research the source of data constituted the majority of the ICSRs (>60% of ICSRs). Literature ICSRs constituted 100% of ICSRs submitted to EV database for 2 DEAs. Only for 1 DEA was the first literature ICSR not indexed in the analyzed bibliographic databases and only for 1 DEA was a literature ICSR not indexed in any database the first report of an adverse event. This analysis confirms the importance of scientific literature as a source of data, in addition to other sources used for the signal management process, which is consistent with other authors’ points of view [5, 9–12].

Klose et al. compared spontaneous and literature ICSRs. They assessed differences in the distribution of ADRs, quality of data, reporting rates of unexpected ADRs from both sources based on data from the German Health Authority (spontaneous ICSRs) and Vigilit database (literature ICSRs). Their study demonstrated the importance of literature ICSRs in addition to other spontaneous ICSRs for a sufficient risk-benefit assessment of medicinal products, and confirmed that none of these sources could be used as a stand-alone system in signal detection [5]. Pontes et al. made similar findings. Despite showing a few examples of literature relevance, such as the case of thalidomide-induced phocomelia, or nifedipine and fatal aplastic anemia, they assessed that the combination of spontaneous and literature ICSRs is necessary for the proper evaluation of drug safety because in some cases the drug’s signal can be detected earlier through regular literature screening and other unexpected adverse events via spontaneous reporting systems [9].

In some cases, it may be reasonable to search information in journals that has a less clinical focus and includes more laboratory-based publications, especially during risk-benefit assessment of medicines that contain a new active substance [8]. The relevance of this type of information was confirmed by the signal for granulocyte macrophage colony-stimulating factor (GM-CSF) and increased risk of viral replication in acquired immune deficiency syndrome (AIDS) patients presented by Pontes et al. [9]. The first concerns about this risk were raised based on in vitro data [13, 14]. This type of safety signal would probably never have been detected based on spontaneous reports as the information came from in vitro studies published in scientific literature.

Based on our research, the systematic review of bibliographic databases, such as Embase or MEDLINE were assessed as the key during the literature screening process. Moreover, to ensure an effective signal management process, attention should be paid to term or text selection, to the approach to records retrieval and the application of limits. Garcelon et al. stated that the literature, specifically MEDLINE, is among the main sources of information used for signal detection [12]. Winnenburg et al. meanwhile confirmed that safety information extracted from MEDLINE is complementary to and not a replacement for other sources of information relevant for a risk-benefit assessment, but for effective literature screening knowledge about database structure is essential to prepare the proper search strategy and accurate retrieval of ADRs [7]. It is worth adding that signal detection based on scientific literature does not cause a delay in detection of a signal, which was confirmed by Shetty et al. They applied information mining to PubMed and discovered that 54% of all detected FDA warnings used literature published before the warnings [15]. This is not consistent with this analysis, where the average delay between first ICSR from a source other than the literature and first literature ICSR was 38 months. This delay may be caused by the long journal review process. This can be also relevant when an author wants to publish a collection of cases. In such situations there could be a long gap between the first and last report. A variety of scientific databases are available and present several differences. Pontes et al. described and compared the databases and search engines relevant for periodic literature screening such as MEDLINE, Embase, Cochrane Library, CINAHL and PubMed, Ovid, ISI Web of Knowledge, ISI Web of Science, Scopus and Google Scholar. They showed the advantages and disadvantages of the most widely used databases. Nevertheless, due to differences such as coverage and accessibility, none of the available databases offers complete sensitivity for the literature screening. Based on this analysis, it is concluded that periodic literature searches should be performed in various carefully selected databases. Decisions about database selection, term or text selection, approach to records retrieval and the application of limits are highly relevant for an effective signal management process. Moreover, an MAH who knows the profile of their medicines can establish the most relevant source of published literature for each product, including the selection of the appropriate scientific database [8].

Based on best knowledge, there has been no previous research comparing the relevance of systematic reviews of journals indexed in bibliographic databases and unindexed journals for the signal management process. It was expected that the impact of unindexed literature (local journals) would be higher than was shown during the analysis of the data, due to its broader local scope and presence, broader data set (analysis of full-text articles instead abstracts) and the several times higher number of locally published journals than of journals indexed in databases. The hypothesis should be put forward that such a low rate of ICSRs from unindexed literature comes from the simple fact that such literature is not adequately screened, and thus not presented properly in the analyzed data. There are almost no required or recommended journal lists available across EU countries for local literature screening, thus each MAH has to assess and justify its own selection of journals without any standard limitation.

It should be also highlighted that 84 of 157 (53.50%) ICSRs created based on information from various types of scientific meeting were not indexed in the analyzed reference databases, which is consistent with the information from appendix 2 of GVP module VI (revision 2) where it is stated that this type of information is a relevant source of data, but is often not available via scientific databases [8].

Moreover, it was noted that publications outside the EEA have a significant impact on the signal management process. For example, 65 literature ICSRs from Japan were not indexed in the analyzed scientific databases.

This study suffers from some limitations. Only those signals which were assessed by the PRAC were analyzed. Only ICSRs from EV were assessed, therefore ICSRs which were collected by an MAH and not sent to EV were not checked. Moreover, the quality of data provided by the literature ICSRs and their significance during the signal assessment step were not verified.

## Conclusion

This study underlines the importance of the scientific literature for the signal management process, in addition to other data sources. Most literature ICSRs from this analysis were created based on scientific journals indexed in scientific databases, therefore it can be concluded that systematic review of scientific databases such as Embase or MEDLINE is highly relevant for the signal management process.
